# Maternally Administered Sustained-Release Naltrexone in Rats Affects Offspring Neurochemistry and Behaviour in Adulthood

**DOI:** 10.1371/journal.pone.0052812

**Published:** 2012-12-26

**Authors:** Waleed O. Farid, Andrew J. Lawrence, Elena V. Krstew, Robert J. Tait, Gary K. Hulse, Sarah A. Dunlop

**Affiliations:** 1 Experimental and Regenerative Neurosciences, School of Animal Biology, The University of Western Australia, Perth, Western Australia, Australia; 2 The Western Australian Institute for Medical Research, Perth, Western Australia, Australia; 3 Unit for Research and Education in Drugs and Alcohol, School of Psychiatry and Clinical Neurosciences, The University of Western Australia, Perth, Western Australia, Australia; 4 Florey Neuroscience Institutes, The University of Melbourne, Melbourne, Victoria, Australia; 5 Centre for Neuroscience, The University of Melbourne, Melbourne, Victoria, Australia; 6 Centre for Mental Health Research, The Australian National University, Canberra, Australian Capital Territory, Australia; Imperial College London, United Kingdom

## Abstract

Naltrexone is not recommended during pregnancy. However, sustained-release naltrexone implant use in humans has resulted in cases of inadvertent foetal exposure. Here, we used clinically relevant dosing to examine the effects of maternally administered sustained-release naltrexone on the rat brain by examining offspring at birth and in adulthood. Maternal treatment (naltrexone or placebo implant) started before conception and ceased during gestation, birth or weaning. Morphometry was assessed in offspring at birth and adulthood. Adult offspring were evaluated for differences in locomotor behaviour (basal and morphine-induced, 10 mg/kg, s.c.) and opioid neurochemistry, propensity to self-administer morphine and cue-induced drug-seeking after abstinence. Blood analysis confirmed offspring exposure to naltrexone during gestation, birth and weaning. Naltrexone exposure increased litter size and reduced offspring birth-weight but did not alter brain morphometry. Compared to placebo, basal motor activity of naltrexone-exposed adult offspring was lower, yet they showed enhanced development of psychomotor sensitization to morphine. Developmental naltrexone exposure was associated with resistance to morphine-induced down-regulation of striatal preproenkephalin mRNA expression in adulthood. Adult offspring also exhibited greater operant responding for morphine and, in addition, cue-induced drug-seeking was enhanced. Collectively, these data show pronounced effects of developmental naltrexone exposure, some of which persist into adulthood, highlighting the need for follow up of humans that were exposed to naltrexone *in utero*.

## Introduction

Naltrexone is a non-selective opioid receptor antagonist [1], used clinically for persons wanting to abstain from opiates [2] and/or alcohol [3], [4]. However, naltrexone is not recommended during pregnancy [5]. Unfortunately, some pregnant opioid-dependent women have received oral naltrexone [6]. Moreover, with the advent of sustained-release naltrexone preparations and their emerging clinical use, inadvertent foetal naltrexone exposure, particularly around conception, is a genuine possibility [7]. To date, 52 women have become pregnant while being treated with an Australian naltrexone implant (GKH, personal communication).

Limited data from humans treated with oral or sustained-release naltrexone suggest no major adverse neonatal outcomes with respect to birth-weights, APGAR scores and head circumferences [6], [8], [9] but long-term follow-up is lacking. However, animal studies, primarily in rat, show adverse neonatal outcomes, but involved daily subcutaneous injections given directly to postnatal offspring rather than clinically relevant maternal (i.e. indirect) and sustained naltrexone exposure. The primary objective of this study was to use a rat model of maternal administration involving sustained-release of naltrexone throughout gestation and lactation at a clinically-relevant plasma concentration of ∼2–10 ng/ml [7], [10] and to determine outcomes in neonates and adult offspring.

Previous studies show that rat neonates directly receiving low-dose naltrexone (1 mg/kg/day) are growth retarded [11], [12] whereas those exposed to high doses (50 mg/kg/day) have increased birth-weights [13], [14]. We therefore assessed the morphometric impact of our sustained-release maternal naltrexone exposure regime hypothesizing that low-dose sustained-release naltrexone exposure would result in reduced birth-weights.

Furthermore, it has been shown that perinatal exposure to naltrexone (1–50 mg/kg/day) can alter aspects of adult offspring behaviour pertaining to emotionality, exploratory drive and analgesic response to morphine [15], [16], [17], [18]. However, changes to locomotor activity are reported not to occur, and to date, no studies are known to have assessed morphine-induced psychomotor sensitization, an indicator of drug-induced plasticity pertinent to aspects of addiction [19], [20]. Moreover, morphine self-administration experiments have not yet been undertaken in naltrexone-exposed offspring. Nonetheless, prenatal exposure to naltrexone can reduce sensitivity to morphine as indicated by decreased open-field locomotion [18]. We therefore assessed morphine-induced sensitization and intravenous morphine self-administration in maternally exposed adult offspring. Given that prenatal naltrexone exposure reduces the density of µ-opioid receptors in offspring with reduced morphine-induced locomotor activity [18], we analysed opioid neurochemistry, namely μ-opioid receptor [21], [22] as well as mRNA for preproenkephalin (PPE) and preprodynorphin (PPD) [23].

## Materials and Methods

### Animals

Experiments adhered to the Prevention of Cruelty to Animals Act, 1986 and the Australian National Health and Medical Research Council Code of Practice for the Care and Use of Animals for Experimental Purposes in Australia. There were 3 cohorts. Procedures for cohort 1 were approved by the Animal Ethics Committee of the University of Western Australia, Perth, Australia (Approval Number: RA 3 100 423) and the Florey Neuroscience Institutes, Melbourne, Australia (Approval Numbers 05-061). Procedures for cohorts 2 and 3 were approved by the Animal Ethics Committee of the University of Western Australia, Perth, Australia (Approval Number: RA 3 100 618) and the Florey Neuroscience Institutes, Melbourne, Australia (Approval Number: 08-001).

**Figure 1:**
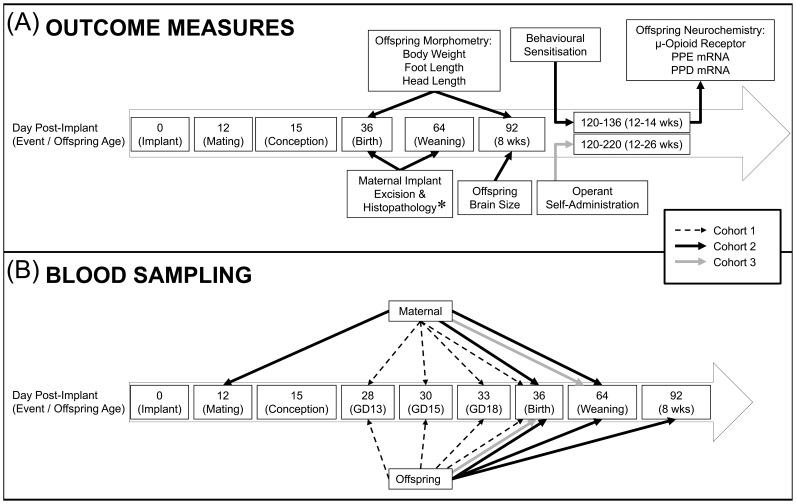
Timelines and overall study design. Timelines and overall study design is shown for all 3 cohorts, including main procedures (i.e. events) and offspring ages (A and B), experimental outcome measures (A), and sampling of blood/amniotic fluid (B). Each cohort can be identified by their respective arrows (cohort 1, black-dashed; cohort 2, black-filled; cohort 3, gray-filled), which provide a guide for the procedures that were undertaken and the approximate end-point. * maternal implant excision and histopathology data published elsewhere [45]. More detailed timelines for the behavioural sensitization and operant self-administration experiments are presented in Figures 2 and 3, respectively.

Three cohorts (Figure 1) of Sprague-Dawley rats (Animal Resource Centre, Murdoch University, Australia) were housed in pairs or threes (23°C; 12-h light/dark cycle) with food/water available *ad libitum*. All females were nulliparous and 9–11 weeks of age upon arrival. In cohort 1, there were 16 dams (180–270 g), in cohort 2 there were 12/treatment group (197–315 g) and in cohort 3 there were 6/treatment group (240–290 g). Ten males (330–460 g) were used for husbandry.

### Drugs

Morphine hydrochloride: Glaxo, Australia, in sterile 0.9% saline. Ketamine: Parnell Laboratories, Australia; meloxicam: Boeringher Ingleheim, Germany; neomycin sulfate: Delta Veterinary Laboratories, Australia; and carprofen: Norbrook Laboratories, Australia. Heparinised saline was diluted at 90 IU/ml.

Custom-sized naltrexone implants comprised the same formulation as for humans (naltrexone-loaded poly[trans-3,6-dimethyl-1,4-dioxyane-2,5-dione] (DL-lactide) microspheres, average molecular weight 700,000 Mv, inherent viscosity 0.5 dl/g, specific rotation of –2° to +2°) compressed into a tablet (total weight: 50 mg; 25 mg naltrexone, 25 mg poly[DL-lactide]; 5 mm diameter × 2 mm height) surrounded by a single coat of poly(DL-lactide) [24]. Placebo implants had the same dimensions and contained 50 mg of poly(DL-lactide) microspheres. All implants were sterilised using irradiation (range 25–40 KGy). Previous *in vitro* (data not shown) and *in vivo* experiments [24] demonstrate that the custom-sized implant used here has similar release rates (0.4–0.8%/day) and plasma levels (2–10 ng/ml) to that measured during their use in humans [10], [25].

### General Procedures

Three cohorts were used in the current study to evaluate the extent of naltrexone exposure in offspring and dams during pregnancy (cohort 1) and before/after pregnancy (cohorts 2 and 3), evaluate gross anatomical outcomes in offspring at birth and adulthood and changes in neurochemistry in adulthood (cohort 2), as well as evaluate behavioural outcomes in adult offspring (cohorts 2 and 3). An overview of the experimental design and timing of events/outcome measures for cohorts 1, 2 and 3 is presented in Figure 1A.

#### Surgery

Dams had a single naltrexone or placebo implant inserted subcutaneously under general anaesthesia (2–3% isoflurane). Carprofen (5 mg/kg, s.c.) and neomycin (50 mg/kg, s.c.) were injected post-operatively. Dams in cohort 1 were implanted with naltrexone and those in cohorts 2 and 3 with naltrexone or placebo implants.

#### Blood sampling and analysis

Blood was collected into lithium-heparinised tubes and frozen at −80°C. For cohort 1, maternal blood was obtained from a lateral tail vein [26] and samples from foetal/neonatal rats by jugular incision, pooling from each litter. Amniotic fluid was also pooled. All other maternal and offspring samples (cohorts 1, 2 and 3) were obtained by incising the atrium after terminal anaesthesia. The concentrations of free naltrexone and 6,β-naltrexol in blood and amniotic fluid samples were determined (Perth Chemistry Centre, Australia) by liquid-chromatography/mass-spectrometry (LCMS) [27]. Reporting limits were 0.2 ng/ml for naltrexone and 0.1 ng/ml for 6,β-naltrexol.

#### Timed mating

Initiation of timed mating took place 12 days after surgical insertion of implants. Timed-matings were undertaken by placement of nulliparous rat dams with 2–3 males overnight; mating was confirmed the following morning by the presence of spermatozoa in vaginal smears. In most cases, conception occurred within 3–5 days of initial presentation to males.

### Cohort 1

#### Naltrexone and 6, β-naltrexol levels during pregnancy

All 16 dams received a naltrexone implant, were mated, and sampled at either gestation day 13, 15 18 or at birth, following euthanasia (n = 4 per time-point). Offspring blood was collected at gestation day 18 and at birth, and was pooled into one sample per litter. At gestation days 13 and 15, amniotic fluid was collected and also pooled into a single sample for each litter (n = 4 per time-point). Naltrexone and 6,β-naltrexol levels in all samples were measured as above. The sampling time-points for maternal rats and offspring from cohorts 1, 2 and 3 are summarised in Figure 1B.

### Cohort 2

#### Naltrexone and 6, β-naltrexol levels before conception, at birth and at weaning

Cohort 2 females were randomly allocated to receive either a naltrexone or placebo implant, were impregnated and gave birth (n = 12/treatment group). Blood was obtained from all maternal rats 1 day prior to mating and at birth. Offspring were either euthanised, sampled for blood and examined at birth (from 6 maternal rats per group), or were raised to 4 weeks, weaned, and raised until 10 weeks (using the remaining 6 dams per group), whereupon they were transferred to the Florey Neurosciences Institute, for evaluation of locomotor behaviour and neurochemistry. Maternal rats fostering these offspring had a blood sample taken at weaning. Individual blood samples were collected from one male and one female from each litter at weaning and at 8 weeks.

#### Body and brain morphometry

Duration of pregnancy, litter size and gender were recorded. At birth and 8 weeks, offspring body-weight was recorded and heel-toe, occipital-snout and biparietal lengths were measured using vernier callipers. At 8 weeks, one male and female per litter were perfused transcardially with heparinised saline (5000 IU/L), followed by 250 ml 4% paraformaldehyde in 0.2 M phosphate buffer (pH 7.2–7.4) for brain morphometry. Brain weight was recorded and cerebrum length, width, height, as well as brain length (i.e. cerebrum+cerebellum) were measured using vernier callipers, and brain volume was calculated by water displacement as described previously [28], [29], [30].

#### Behavioural sensitization

Upon arrival of offspring at the Florey Neuroscience Institutes, animals were acclimatised for 2 weeks and no procedures were undertaken. At 12–14 weeks of age, sensitization to locomotor responses induced by repeated morphine treatment was performed as previously described [31], [32]. Briefly, adult male and female offspring were placed individually in photo-optic locomotor cells (Truscan Photobeam; Coulbourn Instruments, Allentown, PA, USA) in a low luminosity (20 lux), controlled environment, for 60 minutes per day for 3 consecutive days. Vertical (rearing) and horizontal (ambulatory) movements were measured by optic sensor beams.

**Figure 2:**
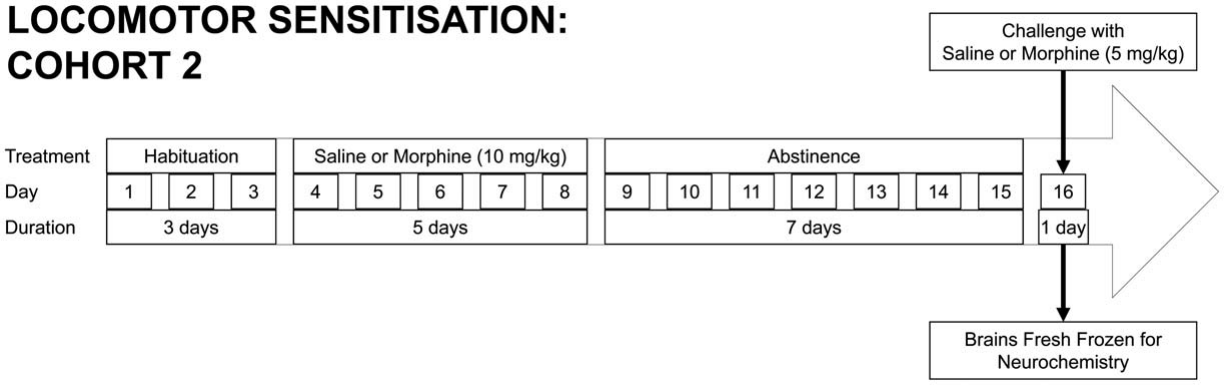
Timeline and paradigm for behavioural sensitization experiment: offspring from cohort 2. Treatments are shown (habituation, daily injection with morphine [10 mg/kg] or saline, period of abstinence, challenge with morphine [5 mg/kg] or saline injection, brains fresh frozen for neurochemistry experiments), with corresponding day since start of locomotor sensitization testing, and number of days for each treatment (i.e. duration).

Testing proceeded as follows. Habituation to the test environment was established using 3 daily 60 min sessions within locomotor chambers. On days 4–8, rats were placed in the locomotor chambers 30 min after an injection of saline (1 ml/kg) or morphine (10 mg/kg, s.c.) and activity recorded for 60 min. From days 9–15, animals underwent a period of home-cage abstinence (i.e. they received no injections). On day 16, rats were challenged with saline or morphine (5 mg/kg) and returned to the locomotor chambers (Figure 2). Treatment groups were: saline-saline, (saline: days 4–8 and 16, SS); saline-morphine (SM); morphine-morphine (MM).

For these experiments, we did not observe differences between males and females. Therefore, for logistical reasons relating to Animal Ethics approval (i.e. minimising animal numbers), only males were used for the subsequent neurochemical and operant self-administration experiments.

#### Neurochemistry

At 14 weeks, after behavioural sensitization testing (∼3 h after final treatment), male rats were terminally anaesthetised (pentobarbitone, 80 mg/kg, i.p.) and brains removed and frozen. Serial sagittal brain sections (4.2–3.4 mm lateral to the mid-sagittal sinus, 14 µm; [33] were thaw-mounted onto slides and stored at −80°C.

[^125^I]DAla^2^,*N*-Me-Phe^4^,Met(*O*)^5^-ol enkephalin (FK33,824) was used to label high affinity µ-opioid receptors [34], [35]. Slides were incubated in 50 mM Tris-HCl (pH 7.4; 0.1% bovine serum albumin, 0.1 nM [^125^I]FK33,824, 2000 Ci/mmol) for 60 min. Non-specific binding was determined by addition of 10 µM naloxone to the incubation medium. Slides were washed, dried and apposed to Biomax AR film (Kodak XAR-5) with [^14^C]microscales. Films were developed automatically (100 Plus Automatic x-ray film processor: All-Pro Imaging).

Expression of PPE and PPD mRNA has previously been described [36]. Oligonucleotides were 3′-end labelled with [α^33^P]d-adenosine triphosphate using terminal deoxynucleotidyl transferase. Labelled probes (1 pg/µl, 100 µl) and applied to adjacent sections in hybridisation buffer (50% formamide, 4×saline sodium citrate, pH 7.0, 10% dextran sulfate). Controls used a 100-fold molar excess of unlabelled oligonucleotide. Sections were hybridised in sealed humidified chambers at 42°C overnight, rinsed (1×saline sodium citrate, 1 h, 55°C), dehydrated in ethanol and air-dried. Dry sections were apposed to x-ray film with [^14^C]microscales and then developed.

Autoradiographs showing binding of radioligand to µ-opioid receptor, and binding of oligonucleotide probes to PPE and PPD mRNA, were captured using a Sony XC-77CE CD Video Camera with an attached Nikon Micro-Nikkor 55 mm lens. Sufficient contrast to background intensity allowed manual delineation of brain regions [33]. Tissue was analysed with an MCID M4 image analysis system under constant illumination; *Scion Image* software was used to convert optical densities to radioactivity per unit area (disintegrations per minute per millimeter squared; DPM/mm^2^) by means of a calibration curve derived from autoradiograms of the simultaneously-apposed [^14^C]microscales.

### Cohort 3

#### Naltrexone and 6, β-naltrexol levels at birth and at weaning

Dams from cohort 3 were randomly allocated to receive either a naltrexone or placebo implant, were mated and gave birth. At birth, litters were reduced to 10 (5 males and 5 females) and culled neonates from each litter had blood pooled into one sample per litter. Surviving offspring were weaned at 4 weeks, at which point maternal blood was collected, and offspring were reared until 10 weeks of age, whereupon they were transported to the Florey Neuroscience Institutes for assessment for operant self-administration of morphine. Upon arrival, animals were acclimatised for 2 weeks and no procedures undertaken.

#### Operant self-administration

Self-administration of intravenous morphine (0.1 or 0.3 mg/kg/infusion) was assessed using operant chambers (Med Associates, USA) with two levers. Rats were connected *via* a jugular cannula to an intravenous line (polyethylene, inner diameter (ID): 0.6 mm, outer diameter (OD): 1.2 mm) connected to a 22-gauge swivel (Instech Solomon, USA) and a syringe held in an infusion pump (PHM-100SVA; Med Associates) with Bcoex-T22 tubing (Tygon, ID 0.6 mm, OD: 1.6 mm; Instech Soloman). A light (conditioned stimulus, CS) came on for 20 s, contingent with an active lever press and reward delivery. In addition, a drop of vanilla essence provided an olfactory cue (S+) for the location of the active lever. The chambers were housed in sound attenuated boxes and ventilated with fans. Med-PC IV software (Med Associates) was used to record lever presses.

The cannulae consisted of a 10 cm length of silastic tubing (inner diameter 0.635 mm, outer diameter 1.194 mm, Dow Corning Corporation, Midland, MI, USA) sleeved over a 22-gauge cannula (Plastics One, Roanoke, VA, USA). All cannulae were autoclaved. At 12 weeks, rats were anaesthetised (2–3% isoflurane) and cannulae implanted into the jugular vein and flushed with 0.15 ml of antibiotic (1.2 mg trimethoprim and 6 mg sulfadoxin) and heparinised saline. Meloxicam (0.75 mg, i.p.) was given for pain relief. The day after surgery, cannulae were flushed with heparinised antibiotic; thereafter, cannulae were flushed twice daily with heparinised saline. Starting on the day of cannulation, rat daily food intake was restricted to 15 g as this is known to facilitate self-administration of abused drugs [37], [38], [39], [40], [41].

After 3 days of recovery in the home cage, rats underwent operant self-administration training to respond for oral sucrose (5%) at a fixed-ratio of 2 (FR2) schedule. Each of these training sessions were 15 h in duration and took place once daily until lever discrimination was demonstrated (after 3 days in most cases). Subsequently, rats were connected to morphine *via* the jugular cannula to the intravenous line. The injection volume was 48 µl per infusion and duration of injection was 2.3 s. A maximum of 50 drug infusions was set, with a 20-s timeout period after an infusion. All post-training sessions (except for cue-induced drug-seeking session at the very end) were 2 h in length, unless 50 infusions were obtained, and were held just before the dark phase of the photoperiod. The active lever remained the same for both oral sucrose training and intravenous self-administration of morphine experiments, as did the position of the olfactory cue and contingent light stimulus.

**Figure 3:**
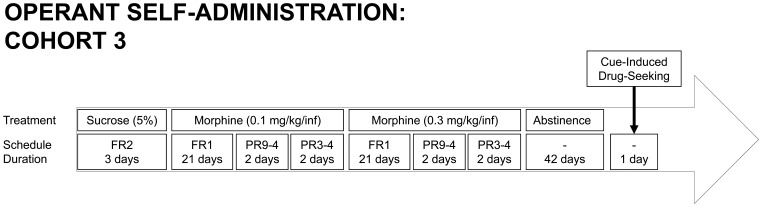
Timeline and paradigm for operant self-administration experiment: offspring from cohort 3. Treatments are shown (oral sucrose training, morphine self-administration at 0.1 mg/kg/infusion dose, then at 0.3 mg/kg/infusion dose, period of abstinence, cue-induced drug-seeking), with corresponding schedule of reinforcement (FR1 and FR2, fixed ratio of 1 and 2, respectively; PR9-4 and PR3-4, progressive ratio 9-4 and 3–4, respectively [see Table 1]; no schedule of reinforcement represented by ‘–’), and corresponding number of days for each treatment schedule (i.e. duration).

Rats responded for intravenous morphine (0.3 mg/kg/infusion) at a fixed-ratio of 1 (FR1) schedule (3 weeks), with subsequent assessment on a progressive-ratio (PR) 9–4 schedule [42] for 2 days, then PR3-4 [43] for 2 days (Table 1). The following day, the rats were returned to FR1 as before, but using a morphine dose of 0.1 mg/kg/infusion; the order and durations for each phase of testing were the same as for the higher morphine dose. Subsequently, rats remained drug-free in the home cage for 6 weeks. Then, drug-seeking was assessed under extinction conditions (FR1 response resulted in CS but no infusion of morphine) for 1 h. An overview of this experimental paradigm of different responding schedules is presented as a timeline in Figure 3.

**Table 1:** Instrumental requirement for PR9-4 and PR3-4 schedules.

Infusion Step Number	1	2	3	4	5	6	7	8	9	10	11	12	13	14	15
Lever Response Requirement, PR9-4	1	1	1	2	2	2	3	3	4	4	5	5	6	6	7
Lever Response Requirement, PR3-4	1	2	4	6	9	12	15	20	25	32	40	50	62	77	95

Lever response requirement values indicate the number of lever presses necessary for the acquisition of each subsequent infusion (infusion step number). Progressive increases vary between the two schedules such that for the 10^th^ infusion, 4 lever presses will result in an infusion using the PR9-4 schedule [42], whereas 32 lever presses are required for the 10^th^ infusion using PR3-4 schedule [43]. Accordingly, the PR3-4 schedule is deemed more difficult as it progressively requires a greater lever response for an infusion than the PR9-4 schedule. Rat responding for morphine was assessed in the current study using both schedules.

The patency of the cannulae was evaluated periodically using ketamine (5 mg). If loss of righting reflex was not apparent within 3 s of infusion, the rat was excluded. In addition, rats were excluded if any cannula blockage or leakage was identified during daily flushing. During assessment of FR1 responding for morphine, criteria were set at a lever discrimination of ≥65% and <25% variation between the numbers of infusions over 3 days.

### Statistical Analyses

Unless otherwise indicated, statistical analysis was undertaken using *SPSS for Windows* and two-tailed tests. Results for free naltrexone and 6,β-naltrexone, in blood or amniotic fluid, were examined with Pearson Correlation coefficient between maternal and offspring levels, stage of pregnancy and time post-implant. Where data failed tests of normality, a Kruskal-Wallis nonparametric ANOVA was employed. Results are for male and females combined unless mentioned otherwise.

Observational and morphometric data were evaluated by one-way ANOVA because violations of the underlying data prevented the use of multiple analysis of variance; accordingly, to reduce the potential for Type 1 errors [44], stringent alpha levels of *p*<0.0125 and *p*<0.008 were used for measures of body and brain morphometry, respectively. Morphometric data are reported separately for male and female offspring (Table 2).

**Table 2:** Cohort 2 offspring body and brain morphometric measurements following maternal treatment with naltrexone or placebo implants.

	DRUG GROUP	
ASSESSED PARAMETERS	Placebo	Naltrexone
**Body Parameters at Birth**	n = 60	n = 70
Body Weight (g)	6.09±0.08	5.42±0.09***
Heel-to-toe length (mm)	9.17±0.09	8.98±0.10
Occipital-to-snout length (mm)	18.21±0.12	18.08±0.13
Biparietal length (mm)	10.54±0.08	10.29±0.09
**Body Parameters in Adulthood**	n = 57	n = 74
Body Weight (g)	250.51±2.99	256.26±2.61
Heel-to-toe length (mm)	43.06±0.17	43.40±0.15
Occipital-to-snout length (mm)	56.96±0.34	55.66±0.30†
Biparietal length (mm)	24.40±0.14	24.21±0.12
**Brain Parameters in Adult Males**	n = 29	n = 37
Brain Weight (g)	1.92±0.06	1.93±0.04
Brain Volume (cm3)	1.84±0.05	1.86±0.04
Brain Length (mm)	19.99±0.13	20.02±0.12
Cerebral Length (mm)	15.35±0.19	15.17±0.15
Cerebral Height (mm)	10.11±0.19	10.25±0.09
Cerebral Width (mm)	15.74±0.16	15.55±0.12
**Brain Parameters in Adult Females**	n = 28	n = 37
Brain Weight (g)	1.79±0.02§	1.76±0.01§
Brain Volume (cm3)	1.72±0.02§	1.70±0.01§
Brain Length (mm)	19.48±0.13§	19.45±0.06§
Cerebral Length (mm)	15.02±0.18	14.87±0.19
Cerebral Height (mm)	9.83±0.08§	9.85±0.12§
Cerebral Width (mm)	15.51±0.15	15.25±0.12

Morphometric effects of maternal treatments during pregnancy and/or weaning on offspring body parameters (body weight, foot and head lengths), at birth and in adulthood (at 8 weeks-of-age). Morphometric effects on brain parameters (brain [cerebral+cerebellar] weight, volume and length, and cerebral length, height and width) are shown for adult male and female offspring and all values represent the mean ± SEM. Using one-way ANOVA: *** *p*<0.001, significant difference for naltrexone versus placebo; **†** *p* = 0.04, trend for difference between naltrexone versus placebo; § *p*<0.01, significant difference for male versus female (within naltrexone or placebo group).

Data from locomotor studies were analysed using a two-way ANOVA, with drug treatment and exposure duration as factors, and with *post-hoc* Student-Newman-Keuls analysis. To evaluate overall differences in activity between treatment groups, a generalised linear model for repeated measures was used.

Neurochemistry data (from examination of only male brains) were incorporated into generalised linear mixed models for statistical analysis using *SAS*; significance and interactions were assessed between the following fixed factors: maternal drug exposure (naltrexone and placebo); duration of morphine exposure; and ligand ([^125^I]FK33,824)/probe ([^33^P]preproenkephalin, [^33^P]preprodynorphin).

Operant self-administration data were obtained for male offspring only. For time course analyses (PR and drug-seeking cumulative responses), and analyses of the effect of implant treatment and dose on morphine self-administration, a two-way ANOVA was performed. Comparisons of the PR data between the offspring groups were analysed using an unpaired t-test. Drug-seeking data were analysed by one-way ANOVA, with Student-Newman-Keuls post-tests. Differences were deemed significant if *p*<0.05, and for pair-wise comparisons, if *p*<0.05 after adjustments using the Dunn-Sidák procedure.

## Results

### General Observations

Data from cohort 2 offspring were combined for analysis of litter size, length of gestation and gender ratio. In cohort 2, there were 10 naltrexone-exposed litters and 10 placebo-exposed litters which, respectively, produced 144, and 117 live pups. There were ∼23% more pups per litter following maternal naltrexone treatment compared to placebo (*F*_(1,19)_ = 13.53; *p*<0.01; Table 3). There was no significant effect of drug treatment on term of pregnancy (*F*_(1,19)_ = 2.70; *p*>0.05; Table 3), nor in gender ratio at birth (χ^2^_(1,19)_ = 3.29; *p*>0.05; Table 3).

**Table 3:** The effects of maternal naltrexone, or placebo, implants on offspring.

		Gender ratio:			
Drug group	Litters: n	Male n (%)	Female n (%)	Litter size: mean (StdDev)	Term (days): median (IQR)
Placebo	10	71 (61)	46 (39)	11.7 (2.0)	21 (21–22)
Naltrexone	10	72 (50)	72 (50)	14.4 (1.2)**	21 (21–21)

The effects of maternal implant treatments during pregnancy on total litter size (observed live and still births), sex ratio of litters and term of pregnancy. Term refers to the length of pregnancy, measured in days. IQR = inter-quartile range. The mean and standard deviation (StdDev) are also shown (one-way ANOVA: ** *p*<0.01, naltrexone versus placebo).

### Naltrexone and 6, β-naltrexol Levels in Dams and Offspring

In both dams and offspring, free 6,β-naltrexol blood-levels were low with many below the limit of detection (>0.1 ng/mg) and a maximum blood concentration of 0.3 ng/ml found in two maternal samples. Nevertheless, a positive correlation existed between naltrexone and 6,β-naltrexol maternal levels: *r* <0.01, N = 94 (data not shown).

**Figure 4:**
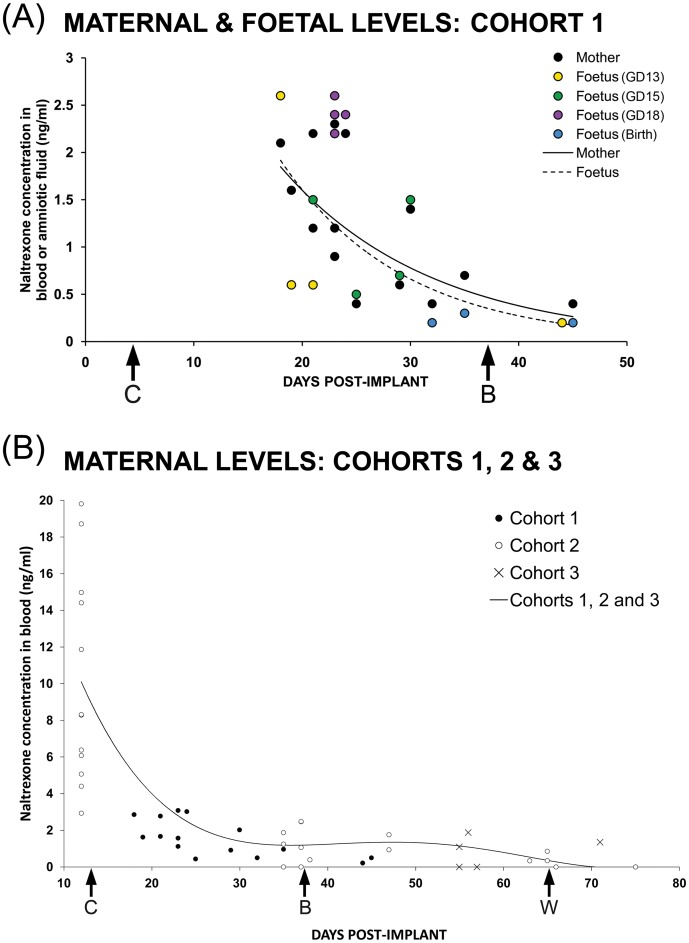
Free naltrexone levels in dams and offspring with naltrexone implant. Temporal profiles of free naltrexone concentration are shown in offspring blood or amniotic fluid (A, cohort 1) or maternal blood (A and B, cohorts 1, 2 & 3.) after maternal administration with a naltrexone implant (25 mg, s.c.). A*:* Temporal profiles of free naltrexone concentration in offspring blood (GD18 [purple] and Birth [blue]) or amniotic fluid (GD13 [yellow] and GD15 [green]) or maternal blood (black) after maternal administration with a naltrexone implant (25 mg, s.c.). Cohort 1 maternal and offspring rats were sampled throughout gestation. Exponential lines of best fit are shown for maternal rats (y = 6.76e^−0.07x^, filled line) and offspring (y = 9.45e^−0.09x^, dashed line). Offspring levels correlated with maternal levels (Pearson Correlation [ = 0.75] significant at 0.001 level; N = 15); and both maternal (*p*<0.01; R^2^ = 0.62) and offspring (*p*<0.05; R^2^ = 0.55) levels correlated with the number of days post-implant. To allow appropriate comparison with offspring, unadjusted maternal data are presented here. C: conception; GD: gestation day; B: birth. B*:* Maternal naltrexone-blood levels prior to conception, at birth and at weaning from cohorts 1 (closed circles), 2 (open circles) and 3 (crosses) were associated with days post-implant (*p*<0.01, N = 54; mean ± SEM: 2.86±0.62 ng/ml). A polynomial line of best fit indicated a moderate level of association (R^2^ = 0.68). C: conception, B: birth, W: weaning.

Free naltrexone was quantified in maternal and offspring blood samples, as well as in amniotic fluid (cohort 1). The weight-adjusted naltrexone concentration in maternal blood was 1.19±0.19 ng/ml; raw offspring naltrexone concentration in blood or amniotic fluid was 1.23±0.25 ng/ml. Further analysis revealed that offspring levels correlated with maternal levels: *r* <0.001, N = 15 (Figure 4A). Furthermore, both maternal (*p*<0.01) and offspring (*p*<0.05) levels correlated with time post-implant (Figure 4A).

For cohort 2, naltrexone was detected in maternal and offspring blood at birth and at weaning (postnatal day 28). Naltrexone was not detected at postnatal day 56 (adulthood). In some offspring at weaning, naltrexone was not detected (n = 8) and these data were excluded from the calculation of means. Unadjusted maternal naltrexone-blood levels are reported elsewhere [45]. Here, we report weight-adjusted maternal mean ± SEM, which declined from 9.74±1.59 ng/ml (n = 12) at 1 day prior to conception to 1.18±0.50 ng/ml (n = 5) at <24 h of birth, 1.18±0.33 ng/ml (n = 5) at postnatal day 3 and 0.30±0.15 ng/ml (n = 5) at weaning. Offspring levels (mean ± S.E.M) were 0.99±0.27 (n = 5) at <24 h of birth and 0.34±0.00 ng/ml (n = 3) at weaning.

At weaning, the weight-adjusted mean ± SEM for free naltrexone in blood from implanted females (cohort 3) was 0.95±0.32 ng/ml (n = 6). Raw data for naltrexone in blood from offspring culled at birth (pooled into one sample per litter) was 3.17±1.65 ng/ml. Weight-adjusted maternal values for free naltrexone-blood concentration with respect to the number of days post-implant are represented for all mothers (cohorts 1, 2 and 3) in Figure 4B. Maternal blood levels sampled from all 3 cohorts correlated with the number of days post-implant (*r* <0.01, N = 54; Figure 4B). Levels after the initial sample taken at preconception (12 days post-implant) also correlated with days post implant (*r* <0.01, N = 42; mean ± SEM: 0.90±0.12 ng/ml; Figure 4B).

### Body and Brain Morphometry

Total litter size, as well as maternal weights from each group, were used as covariates. Covariates appearing in the model of offspring at birth were evaluated at 239.42 (mothers’ weight) and 12.66 (litter size). For offspring evaluated in adulthood, values were 272.22 mothers’ weight and 13.67 for litter size.

After adjusting for covariates, naltrexone had a significant effect on offspring body weight at birth (*F*_(1,129)_ = 14.93; *p*<0.001; Table 2). Estimated marginal means for body weight were 10.9% less in the naltrexone-group compared to that of placebo offspring. There was also a trend for gender to impact on offspring birth-weight (*F*_(1,129)_ = 3.81; *p* = 0.05; Table 2), with males 3.9% heavier than females. Both male and female pups in the naltrexone group had lower birth-weights than placebo counterparts.

In adult offspring, there were no effects from drug treatment on body weight (*F*_(1,130)_ = 1.73; *p*>0.05; Table 2). There was, however, a significant effect of gender on body weight (*F*_(1,130)_ = 14.26; *p*<0.001; Table 2). Naltrexone-exposed adult offspring did not differ significantly from those exposed to placebo with respect to occipital-to-snout length, although planned comparison revealed a trend for naltrexone to affect head size, with a 2.3% reduction in the estimated marginal mean of occipital-to-snout length in naltrexone offspring when compared to placebo (*F*_(1,130)_ = 3.28; *p* = 0.04; Table 2).

For brain morphometry, only adult offspring were examined. Each variable was assessed separately with drug group and gender as fixed factors. None of the measures showed a significant main effect of drug group or a significant group by gender interaction. Each variable showed a significant main effect of gender at the adjusted alpha level except for brain width (*F*_(1,19)_ = 8.10; *p* = 0.01; Table 2), and cerebrum length (*F*_(1,19)_ = 7.70; *p* = 0.01; Table 2); male offspring had larger and heavier brains (Table 2).

### Locomotor Activity and Behavioural Sensitization

**Figure 5:**
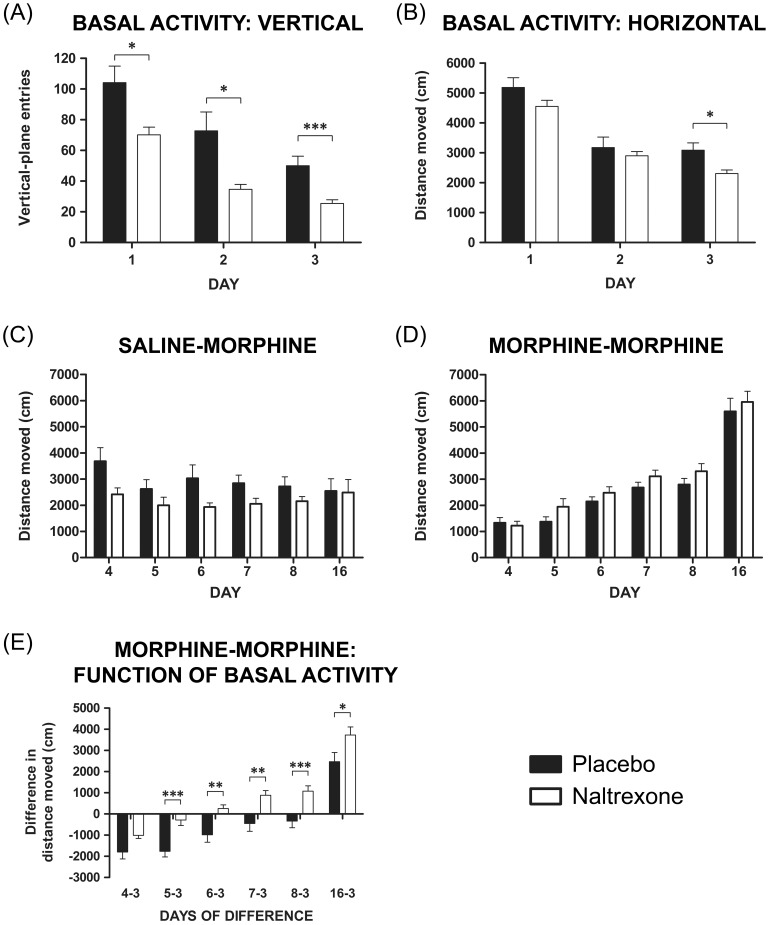
Comparison of locomotor activity between offspring groups in response to a novel environment. Locomotor activity is shown on days 1–3, after saline or morphine administration on days 4–8 (10 mg/kg, s.c.), and after a final morphine challenge on day 16 (5 mg/kg, s.c.). A: Total activity scores in the vertical plane and B: in the horizontal plane, reveal that habituation was greater among naltrexone-exposed offspring. C: Total horizontal locomotor activity did not differ between groups treated with saline and challenged with morphine (saline-morphine). D: Total horizontal locomotor activity did not differ between groups treated with morphine, although sensitization was expressed in both groups after challenge with morphine (morphine-morphine). E: Analysis of data expressing total morphine-induced locomotor activity as a function of basal locomotor activity on day 3 revealed significantly greater activity values for naltrexone-exposed offspring, indicating an increased development of sensitization. Data are expressed as vertical plane entries (A) or horizontal distance moved in centimetres (B, C, D and E) and represent the mean ± SEM (one-way ANOVA: * *p*<0.05, ** *p*<0.01, *** *p*<0.001; days 1–3 (A and B): placebo: n = 37, naltrexone: n = 53; days 4–16 (C): placebo: n = 12, naltrexone: n = 19; days 4–16 (D and E): placebo: n = 20, naltrexone: n = 26).

We did not observe differences between males and females; accordingly, we combined them into a single data set. After initial placement in locomotor chambers, naltrexone-exposed offspring (males and females combined) exhibited fewer vertical plane entries (rearing) compared to placebo-exposed offspring (*F*_(1,88)_ = 5.70; *p*<0.05; 32.6%; Figure 5A) and continued to display significantly less rearing while habituating over days 2 (*F*_(1,88)_ = 6.00; *p*<0.05; 52.3%; Figure 5A) and 3 (*F*_(1,88)_ = 12.90; *p*<0.001; 49.1%; Figure 5A), translating to an overall reduction of basal activity in the vertical plane (*F*_(1,88)_ = 20.45; *p*<0.001; 42.6%). Horizontal plane activity (ambulation) did not differ on day 1 (*F*_(1,88)_ = 1.80; *p*>0.05; Figure 5B), although in naltrexone-exposed offspring, total horizontal activity was significantly attenuated compared to placebo offspring by day 3 for males and females combined (*F*_(1,88)_ = 6.00; *p*<0.05; 25.3%; Figure 5B).

Given the difference in horizontal activity between treatment groups following habituation by day 3, subsequent data for morphine treatments were calculated as a function of basal activity. While there was no difference between groups on the first day of morphine treatment, from day 4 to 5 (i.e. morphine injection day 1 to 2) there was a significantly greater increase in ambulation of naltrexone-exposed offspring which was 11.5 fold greater than placebo-exposed offspring (726.8±248.6 cm compared to 37.0±204.6 cm; *F*_(1,44)_ = 4.19; *p*<0.05; Figure 5D). From day 5 until the completion of testing (day 16), naltrexone-exposed offspring continued to exhibit significantly greater development of sensitization as a function of basal activity (Figure 5E). This was further validated using a generalised linear model for repeated measures which confirmed 42.7% greater development of sensitization during the induction phase and greater overall ambulation of naltrexone-exposed offspring, in males (*F*_(1,20)_ = 5.68; *p*<0.05), females (*F*_(1,22)_ = 9.12; *p*<0.01), and males and females combined (*F*_(1,44)_ = 14.98; *p*<0.001; Figure 5E). There was no effect of saline injection on locomotor activity with respect to time and, likewise, no difference between groups in response to morphine challenge (day 16) following saline pre-treatment (saline-morphine; *F*_(1,29)_ = 0.29, *p*>0.05; Figure 5*C*).

### Opioid Neurochemistry in Offspring Brains

**Figure 6:**
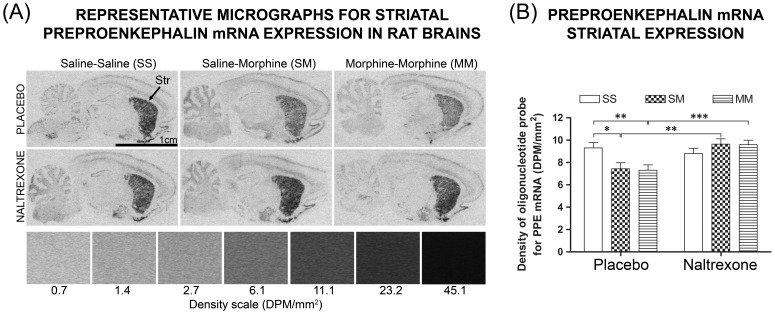
Opioid neurochemistry in brains of offspring. Evaluation of [^33^P]preproenkephalin (PPE) mRNA binding in the striatum (Str) by *in situ* hybridisation histochemistry (ISHH), with comparison between offspring groups (placebo; naltrexone) and drug treatments received during behavioural sensitization (saline-saline, SS; saline-morphine, SM; morphine-morphine, MM). A: Representative autoradiograms showing localisation of [^33^P]PPE mRNA binding sites within midsagittal sections of whole rat brain at approximately 4.0 mm from the midline. Relative PPE mRNA expression within Str presented in accordance with quantitative ISHH (A), which was performed as described in *Materials and Methods*. As indicated by the density scale, the highest binding levels appear as black. B: Quantitative data for PPE mRNA binding in Str are expressed in disintegrations per minute per millimetre squared (DPM/mm^2^) and represent the mean ± SEM. Using generalised linear mixed modelling: * *p*<0.05, ** *p*<0.01, *** *p*<0.001, naltrexone versus placebo by SS versus SM versus MM, naltrexone: n = 14; placebo: n = 17; n≈5/column.

Analysis of brains from male offspring (cohort 2), revealed a two-way interaction between maternal treatment group (i.e. naltrexone or placebo), treatment received during behavioural testing (i.e. saline-saline, saline-morphine, morphine-morphine) and PPE mRNA expression. Namely, reduced expression was associated with placebo offspring given morphine, either as saline-morphine (20.0%; *p* = 0.018) or morphine-morphine (21.4%; *p* = 0.005) compared to expression in morphine-naïve counterparts (saline-saline) (Figure 6A and B). However, no reduction in expression was found in naltrexone-exposed offspring, although striatal PPE mRNA expression was greater in those given morphine (saline-morphine: 29.6%; *p* = 0.002 or morphine-morphine: 31.3%; *p*<0.001) compared to placebo-exposed counterparts (Figure 6*A and B*). This effect on PPE mRNA expression was not found in other brain regions examined (Table 4). No significant differences were found with respect to expression of PPD mRNA or μ-opioid receptor expression (Table 4).

**Table 4:** Mean ± SEM values (DPM/mm^2^) for *in situ* hybridisation histochemistry and autoradiography binding.

MATERNAL TREATMENT GROUP	PLACEBO			NALTREXONE		
Offspring Treatment Group	Saline-Saline	Saline-Morphine	Morphine-Morphine	Saline-Saline	Saline-Morphine	Morphine-Morphine
**PPE mRNA**						
Striatum	9.30±0.48	7.44±0.54*	7.31±0.48**	8.79±0.48	9.64±0.48**†**	9.59±0.40§
Dentate Gyrus	9.32±0.52	10.12±0.58	10.50±0.51	10.36±0.52	9.57±0.51	9.29±0.43
Cortex	8.17±0.42	8.10±0.42	8.69±0.70	7.21±0.54	7.60±0.46	8.15±0.54
**PPD mRNA**						
Striatum	0.65±0.36	0.69±0.40	0.57±0.34	0.56±0.43	0.64±0.35	0.55±0.31
Dentate Gyrus	2.54±0.45	2.61±0.51	2.37±0.44	2.03±0.49	2.00±0.45	2.26±0.38
**µ-Opioid Receptor**						
Striatum	2.73±0.48	2.62±0.55	2.59±0.48	4.02±0.48	3.23±0.48	3.75±0.36
Cortex	1.92±0.06	2.22±0.09	2.20±0.14	2.19±0.11	1.90±0.09	2.21±0.11
Thalamic Nucleus	2.07±0.07	1.91±0.13	1.68±0.11	2.26±0.13	1.86±0.12	1.95±0.15
Medial Geniculate Nucleus	4.59±0.19	4.64±0.35	4.62±0.25	5.33±0.31	4.60±0.25	4.24±0.26
Dorsal Hippocampus	2.50±0.10	2.74±0.11	2.43±0.15	2.61±0.14	2.28±0.11	2.20±0.07
Ventral Hippocampus	7.00±0.31	7.02±0.27	6.44±0.60	7.23±0.32	6.58±0.38	5.97±0.39

Quantitative data for [^33^P]preproenkephalin (PPE) mRNA, [^33^P]preprodynorphin (PPD) mRNA and [^125^I]FK33,824 (µ-opioid receptor) binding in various regions of male rat brains (striatum, dentate gyrus, cortex, thalamic nucleus, medial geniculate nucleus dorsal hippocampus) with comparison between maternal treatment groups (placebo; naltrexone). Using generalised linear mixed modelling, significant differences were found in PPE mRNA binding in striatum: * *p*<0.05, ** *p*<0.01 for significant differences with respect to saline-saline treated offspring from maternal rats implanted with placebo; **†** *p*<0.01 for significant difference with respect to saline-morphine treated offspring from maternal rats implanted with placebo; § *p*<0.001 for significant difference with respect to morphine-morphine treated offspring from maternal rats implanted with placebo; naltrexone: n = 14; placebo: n = 17; n ≈ 5/column.

### Propensity to Self-administer Morphine

#### Fixed-Ratio responding

**Figure 7:**
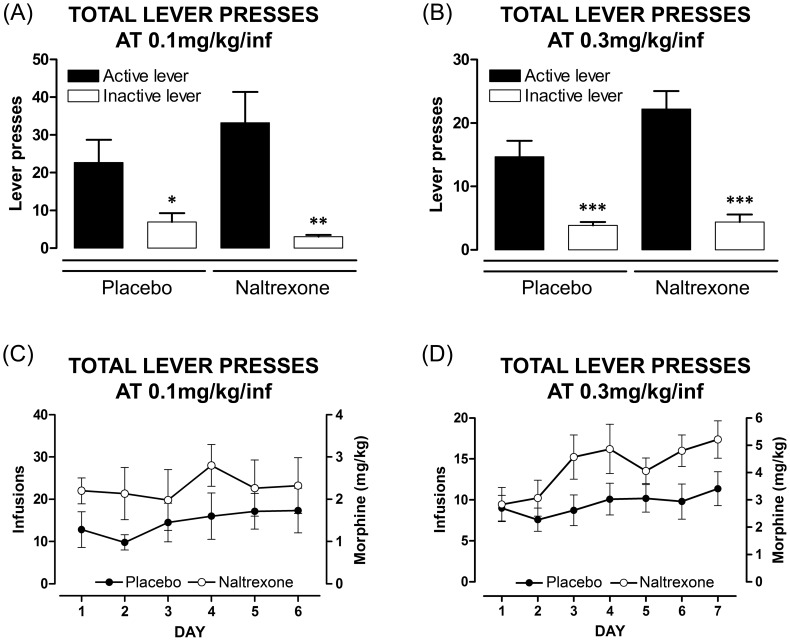
Comparison of naltrexone- and placebo-exposed offspring to fixed ratio morphine self-administration. Morphine self-administration under a fixed-ratio of 1 lever press and acquisition of a stable response. Data are expressed as the number of active *versus* inactive lever responses per 2 h session over 6 days for dose of 0.1 mg/kg/infusion (A), and over 7 days for 0.3 mg/kg/infusion (B), and represent the mean ± SEM (two-way ANOVA, * *p*<0.05, ** *p*<0.01, *** *p*<0.001, compared to the active lever, 0.1 mg/kg/infusion: placebo: n = 6, naltrexone n = 6; 0.3 mg/kg/infusion: placebo: n = 12, naltrexone: n = 9). Data are expressed as number of infusions and amount of morphine (mg/kg) obtained each day over 6 days for dose of 0.1 mg/kg/infusion (*C*) and over 7 days for 0.3 mg/kg/infusion (*D*) represent the mean ± SEM (no difference between groups using two-way ANOVA).

Both naltrexone- and placebo-exposed offspring reliably self-administered morphine (Figure 7). Analysis of mean total data for FR1 responding revealed significant discrimination between the two levers at 0.1 mg/kg/infusion (*F*_(3,20)_ = 35.94, *p*<0.001; Figure 7A). Student-Neuman-Keuls *post hoc* comparisons revealed significant preference for the active lever over the inactive lever in placebo- (*F*_(1,10)_ = 5.97, *p* = 0.035) and naltrexone-exposed rats (*F*_(1,10)_ = 13.54, *p* = 0.004). Significant discrimination was also evident at 0.3 mg/kg/infusion (*F*_(3,38)_ = 18.68, *p*<0.001; Figure 7B) with *post hoc* comparisons revealing significant preference for the active lever over the inactive lever in both placebo- (*F*_(1,22)_ = 17.58, *p*<0.001) and naltrexone-exposed rats (*F*_(1,16)_ = 33.68, *p*<0.001).

Analysis of the number of morphine infusions received over 7 days revealed an overall significantly augmented response by naltrexone-exposed offspring at 0.3 mg/kg/infusion over this period (*F*_(1,15)_ = 6.17, *p* = 0.025). This difference between groups was not observed over a similar period at 0.1 mg/kg/infusion (*F*_(1,7)_ = 0.90, *p*>0.05). At 0.3 mg/kg/infusion, there was a significant escalation in morphine self-administration by naltrexone-offspring, with greater consumption on day 7 compared to day 1 (*t*_(14.63)_ = 2.57, *p* = 0.022; Figure 7D). However, as there was a lack of escalation in self-administration at 0.1 mg/kg/infusion (*t*_(16.16)_ = 0.75, *p*>0.05; Figure 7C), FR1 testing at the lower dose was undertaken for 6 days. The number of timeout responses did not differ between placebo- and naltrexone-exposed offspring at either 0.1 (*F*_(1,10)_ = 0.47, *p*>0.05) or 0.3 mg/kg/infusion (*F*_(1,19)_ = 3.50, *p*>0.05) (data not shown).

#### Progressive-ratio responding

**Figure 8:**
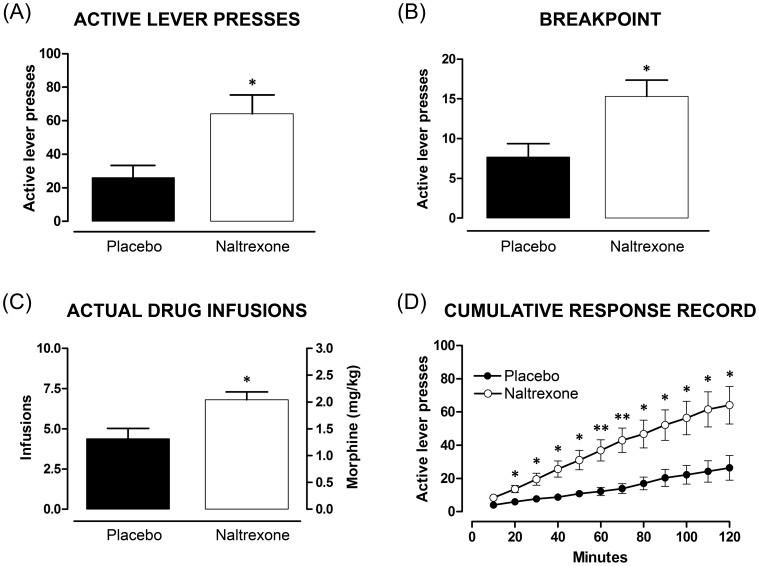
Comparison of naltrexone- and placebo-exposed offspring to progressive ratio morphine self-administration. Morphine self-administration under a progressive ratio (PR) schedule of 3–4 (see Table 1 for response requirement) at a dose of 0.3 mg/kg/infusion. Data from the PR3–4 session are expressed as total active lever presses (A), breakpoint, defined as the final ratio completed within the 2 h session (B), and number of drug infusions (C) and represent the mean ± SEM (unpaired t-test, * *p*<0.05, naltrexone versus placebo (n = 7). D: Cumulative response record for the PR3-4 session divided into 10 minute time bins. Data are expressed as number of active lever presses and represent mean ± SEM (two-way ANOVA, * *p*<0.05, ** *p*<0.01, naltrexone versus placebo (n = 7) for each time bin).

No difference was seen between placebo- and naltrexone-exposed offspring for 0.1 mg/kg/infusion assessed on the PR3-4 schedule with respect to active lever presses (*t*_(10)_ = 0.83, *p*>0.05), breakpoint (*t*_(10)_ = 0.85, *p*>0.05), drug infusions (*t*_(10)_ = 0.83, *p*>0.05), or overall cumulative response (*F*_(1,10)_ = 0.38, *p*>0.05) (data not shown). However, at 0.3 mg/kg/infusion, naltrexone-exposed rats displayed a higher breakpoint compared to placebo offspring on a PR3-4 schedule. Unpaired t-tests revealed a significant difference between offspring groups with respect to the final ratio obtained (breakpoint) (*t*_(12)_ = 2.91, *p* = 0.013; Figure 8B), total number of lever presses for the session (*t*_(12)_ = 2.82, *p* = 0.015; Figure 8A) and number of infusions (*t*_(12)_ = 3.01, *p* = 0.011; Figure 8*C*). Furthermore, when assessing the proportion of rats that reached a ‘true breakpoint’ (when no reinforcer is earned for a 60 min period), none of the 7 naltrexone-exposed rats reached a ‘true breakpoint’ in the 2-h session, whereas 4 of the 7 placebo-exposed rats (57%) did. This is apparent as the clear plateau in the responding of placebo- but not for the naltrexone-exposed offspring when examining cumulative responses (Figure 8D).

Analysis of cumulative responding revealed a significant effect of offspring group (*F*_(1,13)_ = 9.24; *p* = 0.012; Figure 8*D*) and time (*F*_(11,66)_ = 44.06; *p*<0.001), and a significant interaction between the two factors (*F*_(1.36,16.28)_ = 6.89, *p* = 0.012). Dunn-Sidák post test comparisons revealed no difference between placebo- and naltrexone-exposed offspring in the first 10 min time bin (*p*>0.05; Figure 8D) but cumulative responses were significantly greater among naltrexone-exposed offspring, compared to those exposed to placebo, in all remaining time bins (20–120 min: *t* = 7.97, 12.42, 17.61, 22.14, 27.19, 31.58, 32.11, 33.94, 36.64, 39.53 and 39.50, respectively, *p*<0.05 for all except from 60 to 70 min time bins, *p*<0.01; Figure 8D).

For the PR9-4 schedule (data not shown) at 0.3 mg/kg/infusion, groups differed with respect to breakpoint (*t*_(11)_ = 2.29, *p* = 0.043) and infusions (*t*_(11)_ = 2.30, *p* = 0.042). Cumulative responses were significantly greater in naltrexone-, compared to placebo-exposed rats, in the 30, 40 and 90 min time bins (*t* = 2.37, 2.20 and 2.23, respectively, with *p*≤0.05).

#### Cue-induced drug-seeking

**Figure 9:**
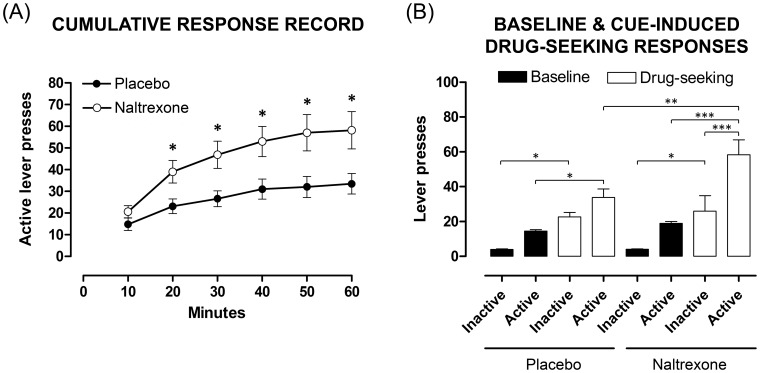
Comparison of naltrexone- and placebo-exposed offspring to cue-induced drug-seeking. Cue-induced drug seeking (cues present but no drug infusions;1 h) after an abstinence period of 6 weeks with comparison between offspring groups (placebo, naltrexone). A: Cumulative response record for cue-induce drug-seeking divided into 10 minute time bins. Data are expressed as number of active lever presses and represent mean ± SEM (two-way ANOVA, * *p*<0.05, naltrexone (n = 9) versus placebo (n = 7) for each time bin). B: Total active and inactive lever presses during the drug-seeking session (represented by white bars, placebo: n = 9, naltrexone: n = 7) as compared to lever presses during 7 days of stable responding under a fixed-ratio 1 (represented by black bars, placebo: n = 12, naltrexone: n = 9). Data expressed as number of lever presses and represent mean ± SEM (one-way ANOVA: * *p*<0.05, ** *p*<0.01, *** *p*<0.001, naltrexone versus placebo).

Both groups exhibited robust cue-conditioned drug-seeking behaviour after a period of abstinence (Figure 9). Nonetheless, overall cumulative responses for the active lever were greater among naltrexone-exposed offspring (*F*_(1.69,23.68)_ = 4.77, *p* = 0.023; Figure 9*A*). Dunn-Sidák *post hoc* test comparisons revealed no difference between placebo- and naltrexone-exposed offspring in the first 10 min time bin (*t*_(14)_ = 1.41, *p*>0.05; Figure 9*A*) but cumulative responses were significantly greater among naltrexone-exposed offspring, compared to placebo, in all remaining time bins (20–60 min: *t* = 2.68, 2.94, 2.73, 2.73 and 2.70, respectively, *p*<0.05; Figure 9*A*). A plateau in responding was evident for both placebo- (40 to 60 min) and naltrexone-exposed offspring (50 to 60 min) (Figure 9A).

Active lever presses were significantly higher on test day (1-h session) compared to baseline responding (2-h session), despite the absence of drug infusions during the session. This significant cue-conditioned drug-seeking was revealed by two-way ANOVA (*F*_(7,66)_ = 16.62, *p*<0.001; Figure 9B), and Student-Newman–Keuls *post hoc* analyses for placebo- (*p* = 0.018; 130%) and naltrexone-exposed offspring (*p*<0.001; 163%), confirming enhanced responding in naltrexone-exposed offspring on the active lever on test day compared to the FR1 schedule. Moreover, compared to placebo, there was augmented active lever responding by naltrexone-exposed offspring on the cue-induced drug-seeking test day versus baseline day (*F*_(1,14)_ = 11.48, *p* = 0.005; 73%; Figure 9B). Furthermore, during the cue-induced drug-seeking session, naltrexone-exposed offspring exhibited significantly greater active lever responding relative to inactive lever presses (*p*<0.001; 124%; Figure 9B); conversely, among placebo rats, responding was comparable between active and inactive levers. There was no difference in latency to first active lever press between naltrexone- and placebo-exposed offspring (*F*_(1,14)_ = 0.046, *p*>0.05).

## Discussion

### Maternal Naltrexone Exposure Induces Behavioural Changes in Adult Offspring

Previous animal studies have shown that prenatal exposure to cocaine, cannabis and morphine leads to an increased propensity to self-administer drugs in adulthood [46], [47], [48] although no effect was observed following prenatal morphine exposure in another study [49]. Nonetheless, the current study is the first to suggest that maternal exposure to sustained-release naltrexone during gestation and lactation confers increased opioid abuse risk in adulthood. The enhanced morphine self-administration in naltrexone-exposed offspring suggests that morphine may have an increased ‘reward value’. Furthermore, increases in breakpoint on a PR schedule suggest increased motivation to obtain the drug [50]. Although both groups showed robust morphine-seeking after 6 weeks of abstinence, naltrexone-exposed offspring exhibited significantly more responses, supporting enhanced relapse-like drug-seeking in naltrexone-treated offspring.

Increased drug-seeking behaviour reflects neuroadaptations that occur with repeated drug exposure [51], [52]. Our findings suggest that developmental opioid receptor antagonism may cause pathophysiological changes that contribute to compulsive drug-seeking behaviour. One possible explanation is that *in utero* naltrexone exposure may negatively impact upon circuits necessary for extinction learning which would also explain an apparently increased motivation to obtain morphine under PR. Interestingly, the endogenous opioid system has been implicated in goal-directed learning as opioid receptor antagonism facilitates the transition from goal-oriented to habitual responding [53]. The present data, therefore, suggest that developmental opioid receptor antagonism may impact upon the pathways implicated in habit-forming. Clearly, a definitive answer for the mechanism behind increased self-administration of opiates and relapse propensity in adult rats exposed to naltrexone *in utero* requires further investigation. One possibility is that striatal enkephalin signalling is altered following naltrexone exposure, as implicated by the neurochemical data. Another possibility is that, rather than naltrexone exposure underlying an increase in reward, the observed increase in operant responding for morphine and cue-induced drug seeking may be a pharmacodynamic effect from naltrexone-induced changes in opioid G-protein receptor coupling or other signalling pathways [54], [55]. Regardless of the mechanism, we suggest that our data argue for long-term follow-up of human cases of foetal naltrexone exposure. For example, the present data provide a strong argument for interrogation of the epigenetic effects of *in utero* naltrexone exposure that may underpin some of the behavioural differences noted in our current study.

Significant differences between naltrexone- and placebo-exposed offspring occurred at the 0.3 mg/kg but not the 0.1 mg/kg morphine dose, consistent with an *inverted U-shaped* dose-response [56], [57], highlighting the importance of using different drug doses. Moreover, the collective data suggest an increased reinforcing strength of morphine in naltrexone-exposed rats that may relate to the relative resistance of striatal enkephalin expression to morphine-induced down-regulation compared to placebo rats.

Naltrexone-exposed offspring exhibited lower vertical activity (rearing) compared to placebos, when first introduced to a novel environment. During subsequent re-exposure to the same environment, less rearing (i.e. habituation) occurred in both groups and remained lower in the naltrexone-exposed group, consistent with other studies [12], [58]. In addition, although similar levels of overall ambulation (horizontal activity) are consistent with other studies [17], [58], [59], we show apparently increased habituation in this plane. Reduced locomotor activity in a novel environment, particularly rearing, could indicate reduced exploratory drive [58] and/or altered motivational systems [60], [61].

Moderate morphine doses in rats produce a biphasic locomotor response [62], [63], which was observed acutely in both naltrexone- and placebo-exposed offspring. Tolerance, the attenuation of a response over a period of chronic drug treatment [64], and sensitization, the increase in a response that arises from repeated drug treatment [62], [65], are different neuroadaptive processes that can occur concomitantly. Repeated morphine treatment produces a decrease in its sedative effects (tolerance) and an increase in its stimulatory effects on locomotion (sensitization) [63], [66]. Changes in vertical and horizontal activity can be used respectively to assess tolerance and sensitization [67]. Drug-induced locomotor sensitization is a robust and well-established indicator of plasticity that may relate to aspects of addiction [19], [20]. Although the expression of sensitization did not differ between groups, naltrexone-exposed offspring showed a greater transition from an initial morphine-induced depressant response to a stimulant response after repeated morphine treatment, suggesting enhanced development of sensitization.

Interestingly, this was mirrored by resistance of striatal enkephalin mRNA expression to down-regulation following morphine treatment compared to placebos. Enkephalin appears to mediate morphine-induced tolerance and sensitization [21], [22], [23] *via* networks known to regulate motor behaviour. Treatment of placebo-exposed offspring with morphine was associated with decreased striatal PPE mRNA expression relative to saline-treated placebo rats. However, this effect did not occur in naltrexone-exposed offspring, indicating that morphine-induced regulation of enkephalin was a normal process [68], [69] not evident following naltrexone-exposure during development. Importantly, this occurred in adult rats that had been free of naltrexone since weaning; indicating that developmental exposure to naltrexone can result in enduring behavioural changes and neurochemical alterations to central opioid systems.

Although an effect was observed for PPE mRNA, no such changes were seen for PPD mRNA or μ-opioid receptor expression. Other studies have shown changes to opioid receptors following naltrexone exposure [70], [71]. For example, in adult mice implanted with 15 mg naltrexone pellets, up-regulation of μ- and δ-opioid receptors was observed after 7 days of naltrexone pre-treatment [72]. Given that up-regulation of μ- and δ-opioid receptors correlate with potency changes of morphine [71], [73] further studies in our model would be interesting to examine whether changes to κ- and/or δ-opioid receptors play a role in the behavioural effects we observed.

### Gross Morphological Changes Following Maternal Naltrexone Exposure are Minimal

Persistent behavioural effects occurred in the absence of substantial morphological changes in adult offspring but were preceded by decreased birth-weights. Because litter size impacts on growth and nutritional intake [74], [75], we incorporated the naltrexone-associated increase in litter size as a covariate and showed that decreases in neonatal body weight occurred independently of litter size. Consistent with our findings, offspring body weight in mice is reduced following low-dose maternal naltrexone [76] and is associated with intermittent postnatal opioid receptor antagonism [77], [78]. Reduced birth-weight and head-size following low maternal and foetal naltrexone exposure (<2 ng/ml) is also consistent with the general inhibitory effects of low-dose opioid receptor antagonism [13], [17], [77], [78], [79], [80], [81], [82]. The trend toward reduced adult offspring head-size, not evident at birth, highlights the need for follow-up in naltrexone-exposed human newborns who appear to be developmentally unaffected [6], [8], [9], [83].

### A Pregnant Rat Model for Sustained-release Naltrexone

The sustained-release naltrexone preparation comprised the same formulation used in humans and yielded comparable release- (∼0.4 mg/kg/day) and blood-levels (∼2–10 ng/ml) [7], [10]. Following intraperitoneal injection in rat, naltrexone undergoes placental transfer [84] but does not remain in pre-weaning offspring blood for more than 2 days after the last injection [85]. Using our customised implant, we have shown transfer of naltrexone to the amniotic fluid, blood and lactate. Humans and rats are also similar with respect to naltrexone’s affinity for opioid receptors [86], [87], plasma protein binding [88] and rate of elimination [89], [90], with a half-life range of 2–14 hours in humans [91], [92], and 4.6–11.4 hours in rats [93], [94].

In relative terms, brain development in rats and humans occurs to a common timetable defined by the time between conception and eye opening (“*caecal period*” rat: 36 days; human: 182 days). When expressed as a percentage of the caecal period, neurodevelopmental milestones start and finish at approximately the same value [95], [96], [97]. Based on caecal period calculations, blood-naltrexone levels were sustained above 1 ng/ml, comparable to levels in humans, for a clinically comparable amount of time (>30 days in rat ≈ 5.5 months in humans). Moreover, the decline in naltrexone levels in maternal rat plasma was similar to the profiles in non-pregnant humans [10], [98] and a single case study of a pregnant woman [7].

Future animal studies investigating the developmental effects of sustained-release naltrexone, particularly on addiction-related behaviours, would provide greater insight in the clinical context if maternal rats were opioid-dependent prior to treatment with naltrexone. Nonetheless, the current study still has much to offer as a clinically-relevant model in that there are a number of potential therapeutic uses for naltrexone in the non-opioid dependent patient, including in the management of alcoholism [3], [4], [99], compulsive gambling [100], [101], [102], [103], multiple sclerosis [104], [105], [106] and obesity [107], [108], [109].

The current study highlights the vulnerability of the developing brain to drug exposure. Indeed, factors such as “imprinting” may be critical at the time of birth. For example, in humans, obstetric administration of opiates, barbiturates or nitrous oxide (>1 hour) during labour and within 10 hours of birth increased the relative risk of offspring subsequently becoming addicted to opiates in adulthood [110], [111], [112]. Our model examined continuous naltrexone exposure during pregnancy and lactation but, in future studies, may be of value to determine whether much shorter critical period/s of exposure also produce effects in adult offspring.

### Conclusion

The current study demonstrates that chronic, low-dose maternal naltrexone delivered *via* a sustained-release implant impacts both behaviour and neurochemistry in adult offspring, but without obvious morphological effects. Our data are consistent with perturbations that are reported to result from naltrexone exposure during development with significant implications for alterations in morphine-induced neuroplasticity, and increased risk of opioid-abuse later in life. Our findings highlight the need for greater information on the safety of using naltrexone during human pregnancy.
